# Clinical utility and cost-effectiveness of bacterial 16S rRNA and targeted PCR based diagnostic testing in a UK microbiology laboratory network

**DOI:** 10.1038/s41598-020-64739-1

**Published:** 2020-05-14

**Authors:** Dinesh Aggarwal, Tanmay Kanitkar, Michael Narouz, Berge S. Azadian, Luke S. P. Moore, Nabeela Mughal

**Affiliations:** 1 Chelsea and Westminster NHS Foundation Trust, 369 Fulham Road, London, SW10 9NH UK; 2 North West London Pathology, Imperial College Healthcare NHS Trust, Fulham Palace Road, London, W6 8RF UK; 3 Imperial College London, Hammersmith Campus, Du Cane Road, London, W12 0NN UK

**Keywords:** Molecular medicine, Diagnosis, Infectious diseases

## Abstract

16S ribosomal-ribonucleic acid polymerase chain reaction (PCR) and targeted PCR aid microbiological diagnosis in culture-negative clinical samples. Despite routine clinical use, there remains a paucity of data on their effectiveness across a variety of clinical sample types, and cost-effectiveness. In this 4 year multicentre retrospective observational study, all clinical samples referred for 16S PCR and/or targeted PCR from a laboratory network serving seven London hospitals were identified. Laboratory, clinical, prescribing, and economic variables were analysed. 78/607 samples were 16S PCR positive; pus samples were most frequently positive (29/84; p < 0.0001), and CSF least (8/149; p = 0.003). 210/607 samples had targeted PCR (361 targets requested across 23 organisms) with 43/361 positive; respiratory samples (13/37; p = 0.01) had the highest detection rate. Molecular diagnostics provided a supportive microbiological diagnosis for 21 patients and a new diagnosis for 58. 14/91 patients with prescribing information available and a positive PCR result had antimicrobial de-escalation. For culture-negative samples, mean cost-per-positive 16S PCR result was £568.37 and £292.84 for targeted PCR, equating to £4041.76 and £1506.03 respectively for one prescription change. 16S PCR is more expensive than targeted PCR, with both assisting in microbiological diagnosis but uncommonly enabling antimicrobial change. Rigorous referral pathways for molecular tests may result in significant fiscal savings.

## Introduction

Molecular diagnostics have significantly enhanced laboratory ability to detect and identify bacteria in clinical samples^1,2^ Whilst bacterial culture is considered the gold standard for microbiological diagnosis, there may be a 24–48 hour delay in providing a result for typical organisms and longer for slow-growing organisms such as *Mycobacteria spp*^3^. Furthermore, false negative culture results may arise from fastidious organisms, non-viable bacteria, or prior use of antimicrobials, potentially affecting patient management.

Two methods of Polymerase Chain Reaction (PCR) are recommended as supplementary tests by the United Kingdom Standards of Microbiology Investigations (SMI); targeted PCR and 16S ribosomal ribonucleic acid (rRNA) PCR^4^. 16S PCR is a pan-bacterial molecular diagnostic test^5,6^, whilst targeted PCR looks for a finite range of organism targets where specific pathogenic organisms are suspected^7^. Identification of causative organisms through targeted PCR or 16S PCR may influence clinical management decisions, serving to support or provide a microbiological diagnosis or impacting antimicrobial prescribing decision-making^8,9^.

The utility of 16S PCR in identifying causative organisms from specific sterile site samples has been demonstrated in multiple isolated clinical syndromes^10–15^. The wider utility of 16S PCR has been evaluated by Rampini *et al*.^16^, who demonstrated a sensitivity and specificity of 42.9% and 100% for culture-negative bacterial infections respectively. This study did not evaluate the functionality of this test based on sample type and we hypothesise this will significantly impact the test’s utility. More recently, Tkadlec *et al*.^9^ published a prospective study on the added value of 16S PCR by sample type, demonstrating additional benefit with joint and heart valve samples. The samples included in this study however were limited and predominantly blood cultures (62%), furthermore it is not a study of culture-negative samples and not reflective of real-world use of this test. The utility of targeted PCR in assisting the diagnosis in other clinical scenarios has been demonstrated, but lack data on whether samples were culture-negative specimens, the cost to the referring laboratories, or clinical utility^2,17^.

We conducted a retrospective observational study to identify all samples referred for 16S PCR and subsequently targeted PCR from a large centralised NHS (National Health Service) clinical laboratory serving seven hospitals and analysed the frequency of positive results based on sample type, the appropriateness of testing, clinical utility in providing diagnoses and enablement of antimicrobial de-escalation, and the cost-per-positive result.

## Methods

### Study design

A retrospective observational study was undertaken to evaluate the utility of 16S PCR and targeted PCR in an NHS laboratory network setting (a centralised laboratory in a hub-and-spoke model). The study was conducted in accordance with the Helsinki declaration and reported in line with STROBE guidelines.

### Setting

North-west London Pathology (NWLP) provides centralised clinical laboratory services to seven London hospitals and over 100 primary healthcare facilities caring for a population of over two and a half million patients. The seven hospitals provide tertiary referral neurosurgery, neurology, cardiothoracic, nephrology, haematology, ophthalmology, vascular, hepatobiliary, orthopaedic, trauma, plastics, burns, paediatric, infectious diseases, and neonatal services. Across all sample types, approximately 850,000 clinical samples for culture and susceptibility are processed annually, resulting in approximately 150,000 clinical isolates per annum back to clinicians. Transit times from referring hospitals to the centralised microbiology laboratory are on average, less than 24 hours. UK SMI standard operating procedures (SOPs) are observed with minor local variation^18^. Microbiological culture is processed in house, with bacterial identification undertaken using matrix assisted laser desorption/ionisation-time of flight (Biotyper, Bruker). Susceptibility testing is undertaken using European Committee on Antimicrobial Susceptibility Testing (EUCAST) breakpoints^19^. The laboratory is accredited through the United Kingdom Accreditation Service. As with many laboratories, targeted PCR for specific bacterial pathogens and 16S PCR is not performed in-house, but referred to third-party providers. NWLP sends samples to Great Ormond Street Hospital, Micropathology Limited, and Public Health England for both tests. These reference laboratories use proprietary in-house methods for extraction and PCR. Samples are only referred for molecular diagnostics on the decision a Medical Microbiologist or Infectious Disease specialist involved in that case. The decision of the Medical Microbiologist or Infectious Diseases specialist to agree to the tests or not was based upon the composite clinical picture and the a priori likelihood of clinical utility from these tests (i.e. testing for non-clinical research purposes was not conducted). Samples may concurrently be sent for further extended culture-based diagnostics (including enrichment broths, and extended fungal, filamentous bacteria, or mycobacterial culture media).

### Data collection

The laboratory information management system (Sunquest v 8.2) was interrogated to identify all samples referred from NWLP for 16S or targeted PCR during four fiscal years, April 2015 to April 2019. Culture results from concurrent samples sent during the clinical episode were reviewed. Patient records were examined to identify prescription impact from positive PCR results. Samples sent just to assist identification of a cultured organism, or those referred but not sent, were excluded from this study. Data was collected using a data collection tool with demographic, laboratory, and clinical fields. Patient data was anonymised at the point of collection.

### Data analysis

Samples sent for 16S PCR and targeted PCR were delineated by sample type, and where positive, the organism identified. Molecular and culture results were compared, and a comparison made in PCR positivity between different sample types. Samples analysed included joint fluid, tissue specimens, CSF, pus, pleural fluid, pericardial fluid, peritoneal fluid, bone marrow, bronchioalveolar lavage (BAL) fluid, blood, vitreous fluid and urine. Statistical analysis was carried out using STATA IC 13 (College Station, TX), with Chi squared (and Fisher’s exact where necessary) tests.

### Definitions

Inappropriate referral for molecular diagnostics was based on samples referred despite culture positivity. Time-to-result was calculated using the time sample was received in the laboratory and when the result was released to clinicians by the Medical Microbiologist. A supportive microbiological diagnosis by molecular diagnostics was a result concordant with another culture sample from the patient. A new microbiological diagnosis was defined as a molecular diagnostic result which identified a new clinically relevant bacterial species (i.e. not previously known from any samples from the patient). A clinically significant antimicrobial change was defined as a prescription change to a more efficacious or narrow-spectrum antimicrobial based on molecular diagnostic result. An alternate diagnosis preferred to the positive 16S PCR or targeted PCR result was noted when documented by the clinical team in the patient record. The cost of sample handling, transport, and processing at the reference laboratories for 16S (£51.50) and targeted PCR (£52.99) samples was derived from billing charges and averaged across the three referral laboratories used.

### Ethics approval and consent to participate

Informed consent was not required as anonymised data was extracted from data collected as part of routine clinical care. Approval was gained for the project as a service evaluation from the Research and Governance Department at North-west London Pathology at Imperial College Healthcare NHS Trust (ref: MCB_002).

## Results

**Figure 1:**
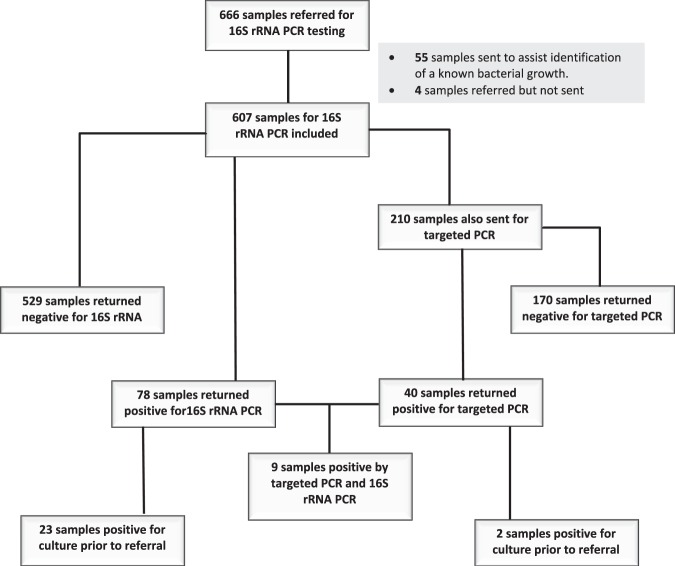
Sample referral and outcome for 16S rRNA PCR and targeted PCR, and number of samples referred with a culture positive result at a London NHS laboratory network, April 2015 and April 2019. abbreviations: ribosomal Ribonucleic Acid (rRNA), Polymerase Chain Reaction (PCR).

Of 666 microbiological samples referred for 16S PCR, 607 were included (Fig. 1). Among those samples sent for 16S PCR, 210 had targeted PCR requested with clinicians looking for 367 bacterial targets corresponding to 23 different species. The mean age of patients from whom these samples were sent was 44 years (range 0–97 years), and 251 (40.7%) were female. The mean referral-to-result time was 13 days (median = 11, range 2–104 days).

### Molecular bacterial detection by sample type

78/607 (12.9%) samples referred for 16S PCR yielded a positive result, while 40/210 samples (19.0%) sent for targeted PCR were positive, but for both molecular tests this varied markedly across sample types (Table 1). For the aggregated samples submitted for molecular diagnostics, a positive targeted PCR result was more likely compared to 16S PCR (p value = 0.04).

**Table 1:** Comparison of 16S rRNA PCR and targeted PCR at a London NHS laboratory network, April 2015 and April 2019.

Targeted PCR				16S PCR			
Sample	Number	Positive	Positivity rate (%)	Number	Positive	Positivity rate (%)	Significant difference between molecular modalities (p)
CSF^a^	62	8	12.9	149	8	5.4	0.11
Pus^b^	18	3	16.7	84	29	34.5	0.23
Tissue/Biopsy^c^	49	8	16.3	131	14	10.7	0.44
Joint Fluid^d^	40	8	20	157	13	8.3	0.06
Peritoneal Fluid	0	0	n/a	9	2	22.2	n/a
Bronchial Alveolar Lavage^e^	6	4	66.7	9	3	33.3	0.46
Pleural Fluid^f^	31	9	29.0	45	5	11.1	0.09
Pericardial Fluid^g^	4	0	0	12	2	16.7	n/a
Vitreous fluid	0	0	n/a	4	0	0	n/a
Blood culture	0	0	n/a	4	2	50	n/a
Bone Marrow	0	0	n/a	2	0	0	n/a
Urine	0	0	n/a	1	0	0	n/a
Total	210	40	19.0	607	78	12.9	**0.04**

Abbreviations: Polymerase Chain Reaction (PCR), Cerebrospinal Fluid (CSF). Combinations of targeted PCRs sent: CSF^a^ (*Streptococcus pneumoniae,* Group A/B *Streptococcal spp., Staphylococcus aureus, Listeria sp., Escherichia coli, Enterobacter sp., Brucella sp., Neisseria sp., Mycoplasma sp., Mycobacteria sp., Leptospira sp. Ureaplasma urealyticum, Tropheryma whipplei*); Pus^b^ (*Streptococcus pneumoniae*, Group A/B *Streptococcal spp., Staphylococcus aureus, Propionibacterium sp., Mycobacterium sp., Actinomyces sp.*); Tissue/Biopsy^c^ (*Streptococcus pneumoniae,* Group A/B.); Joint Fluid^d^ (*Streptococcus pneumoniae*, Group A/B *Streptococcal spp., Staphylococcus aureus, Escherichia coli, Kingella kingae, Neisseria sp., Salmonella sp., Mycobacteria sp.*); Bronchial Alveolar Lavage^e^ (*Streptococcus pneumoniae, Legionella pneumophila*); Pleural Fluid^f^ (*Streptococcus pneumoniae*, Group A/B *Streptococcal spp., Staphylococcus aureus, Haemophilus influenzae, Mycoplasma sp., Mycobacteria sp., Coxiella burnetti*); Pericardial Fluid^g^ (*Streptococcus pneumoniae, Actinomyces sp., Borrelia Burgdorferi*).

For 16S PCR, the sample with the highest detection rate was pus (29/84, 34.5%; p < 0.0001 compared to all other sample types); CSF samples demonstrated the lowest organism detection rate by 16S PCR, with 8/149 positive (5.4%; p = 0.003 compared to all other sample types). Joint fluid was the most commonly referred sample type, resulting in 13/157 (8.3%; p = 0.21 compared to all other sample types) positives; 8/136 (5.9%) positive from native joints, and 5/21 (23.8%) positive from prosthetic joint synovial fluid. Please see Supplementary Tables 1 and 2 for the diversity of sites from which tissue and pus samples were obtained respectively, with corresponding 16S PCR results.

For targeted PCR, 40/210 samples (19.0%) were positive corresponding to a total of 43 bacteria. The highest detection rate by sample type was for respiratory samples (13/37 (35.1%); p = 0.01 compared to all other sample types), with 4/6 (66.7%) BAL fluid and 9/31 (29.0%) pleural fluid positive.

### Molecular bacterial detection by organism type

For 16S PCR, Group A and group B *Streptococcus spp*. (6/78), *Streptococcus spp*. (to genus level only; 6/78) and *Haemophilus sp*. (6/78) were the most common bacterial species detected. 28 different bacterial species were identified with 16S PCR, with 8 isolates not identified beyond *Staphylococcus spp*. or *Streptococcus spp*. genus level. A further 10 positive 16S PCR results identified mixed species and 3 organisms could not be identified to a genus level (Table 2).

**Table 2:** Comparison of microbiological diagnosis by culture, 16S rRNA PCR and targeted PCR at a London NHS laboratory network, April 2015 and April 2019.

Organism	Culture (n = 607)	16S rRNA PCR (n = 607)	Targeted PCR (n = 367)
**Gram Positive Organism**			
*Staphylococcus aureus*	8	1	9
Unidentified *Staphlococcus spp*	0	2	n/a
Coagulase negative *Staphyloccocus spp*	17	3	n/a
*Streptococcus pneumoniae*	0	2	13
Group A/B *Streptococcus spp*	0	6	10
*Streptococcus dysgalactiae*	0	2	n/a
*Streptococcus milleri* group	2	3	n/a
Unidentified *Streptococcus spp*	2	6	n/a
*Enterococcus sp*	3	2	n/a
*Propionibacterium sp*	2	2	2
*Bacillus sp*	0	1	n/a
*Corynebacterium sp*	0	1	n/a
*Dermacoccus sp*	0	1	n/a
*Eubacterium sp*	0	1	n/a
*Gemella sp*	0	1	n/a
*Aerococcus sp*	1	0	n/a
**Gram Negative Organism**			
*Haemophilus sp*	1	6	1
*Pseudomonas sp*	3	3	n/a
*Escherichia coli*	1	3	1
*Klebsiella sp*	0	1	n/a
*Herbaspirillum sp*	0	2	n/a
*Prevotella sp*	1	1	n/a
*Aeromonas sp*	0	1	n/a
*Haematobacter sp*	0	1	n/a
*Neisseria sp*	0	1	0
*Proteus sp*	0	1	n/a
*Ureaplasma sp*	0	1	1
*Achromobacter sp*	1	0	n/a
*Paracoccus sp*	0	1	n/a
*Bartonella sp*	0	0	1
*Chlamydia sp*	0	0	1
*Kingella kingae*	0	0	1
**Anaerobes**			
*Fusobacterium sp*	0	3	n/a
*Aggregatibacter sp*	0	1	n/a
*Actinomyces*	1	0	0
**Other**			
*Mycoplasma sp*	0	5	0
*(Candida albicans)*	1	0	n/a
*Mycobacterium sp*	0	0	3
Unidentified organism	3	3	n/a
Mixed species	4	10	n/a
Total positives	**51**	**78**	**43**

Abbreviations: ribosomal Ribonucleic Acid (rRNA), Polymerase Chain Reaction (PCR)

For targeted PCR, *Streptococcus pneumoniae* (13/44) was the most commonly identified bacteria by targeted PCR. *Streptococcus pneumoniae*, Group A & group B *Streptococcus spp*. and *Staphylococcus aureus* contributed to 32/44 of positive results by targeted PCR, with a total of 9 different species identified (Table 2).

### Clinical utility

**Figure 2:**
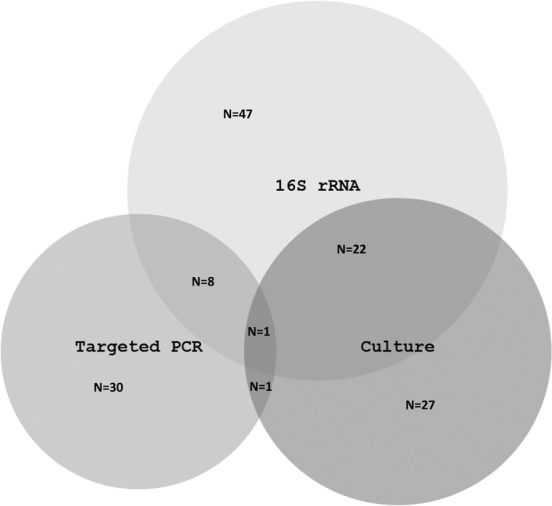
Positive results for 16S rRNA PCR, targeted PCR and microbiological culture among 607 samples referred for molecular diagnostics at a London NHS laboratory network, April 2015 and April 2019. abbreviations: ribosomal Ribonucleic Acid (rRNA), Polymerase Chain Reaction (PCR).

**Figure 3:**
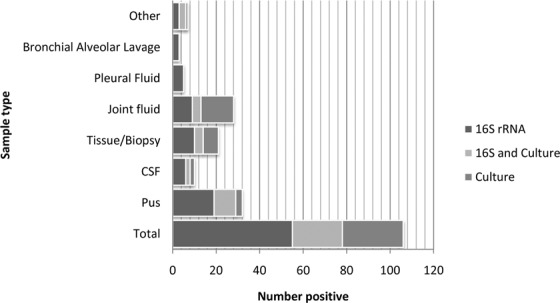
(**a**) Positive results by 16S rRNA PCR and microbiological culture by sample type at a London NHS laboratory network, April 2015 and April 2019. Abbreviations: ribosomal Ribonucleic Acid (rRNA), Polymerase Chain Reaction (PCR), Cerebrospinal Fluid (CSF). (**b**) Positive results by 16S rRNA PCR and targeted PCR by sample type at a London NHS laboratory network, April 2015 and April 2019. Abbreviations: ribosomal Ribonucleic Acid (rRNA), Polymerase Chain Reaction (PCR), Cerebrospinal Fluid (CSF). (**c**) Positive results by targeted PCR and microbiological culture by sample type at a London NHS laboratory network, April 2015 and April 2019. Abbreviations: Polymerase Chain Reaction (PCR), Cerebrospinal Fluid (CSF).

51/607 (8.4%) samples submitted for molecular diagnostics ultimately had a positive culture result (from additional enriched media/prolonged culture etc) prior to receiving the final molecular result. Of these, 23/51 were 16S PCR positive, whilst 28/51 were negative, correlating (for this subset) to a sensitivity of only 45%. 9/210 samples sent for targeted PCR and 16S PCR were positive through both modalities. The use of molecular diagnostics (16S and targeted PCR) provided an additional positive result in 86/607 samples sent (Fig. 2). Figure 3 details this further with a breakdown by sample type.

Molecular diagnostics (either 16S PCR, targeted PCR or a combination of both) provided a supportive microbiological diagnosis in 21/109 (19.3%) and a new microbiological diagnosis in 58/109 (53.2%). 91 samples had associated prescribing information available and of these, a positive molecular diagnostic result translated to a clinically significant antimicrobial change in 14/91 (15.4%) cases. This was not significantly different between a positive 16S PCR 9/64 (14.1%) and targeted PCR 7/36 (19.4%) (p = 0.67). Positive molecular results were thought to represent contaminants and an alternate diagnosis more likely for 12/78 (15.4%) positive 16S PCR results compared to 2/40 (5%) positive targeted PCR results (p = 0.18).

### Cost-effectiveness

For 16S PCR, the cost of testing 607 samples was £31,260.50. Across all sample types, the number needed-to-test to obtain one positive 16S PCR result was 7.78 resulting in an average cost-per-16S PCR positive result of £400.78. If those samples that were ultimately culture-positive are discounted, the number needed-to-test to obtain one positive 16S PCR/culture-negative result rose to 11.0 and the average cost to £568.37. The notable variation in number needed-to-test indicates cost-effectiveness is markedly altered depending on the sample type (Table 3). Among the subset for whom prescribing data was available, the cost for a 16S PCR positive/culture-negative result to impact an antimicrobial prescription equated to £4041.76.

**Table 3:** Comparison of cost-per-positive (£) by sample type for 16S rRNA PCR positive results at a London NHS laboratory network, April 2015 and April 2019.

Samples referred	Number sent	Cost(£)/positive 16S rRNA	Cost(£)/positive 16S rRNA and negative culture
Pus	84	149.17	227.68
CSF	149	959.19	1278.92
Tissue/Biopsy	131	481.89	674.65
Joint Fluid	157	621.96	898.39
Pleural Fluid	45	463.50	463.50
Bronchial Alveolar Lavage	9	154.50	154.50
Other	32	274.67	549.33
All samples	607	400.78	568.37

For targeted PCR, the cost of testing 210 samples was £11,127.00. The number needed-to-test to obtain one positive targeted PCR result was 5.25 resulting in an average cost-per-targeted PCR positive result of £278.20. If those samples that were culture-positive are discounted, the number needed-to-test to obtain one positive targeted PCR/culture-negative result rose to 5.53 and the average cost to £292.84. Similarly to 16S PCR, the marked variation in number needed-to-test correlates to notable variation in cost-effectiveness between sample types (Table 4). Among the subset for whom prescribing data was available, the cost for a targeted PCR positive/culture-negative result to impact an antimicrobial prescription equated to £1506.03.

**Table 4:** Comparison of cost-per-positive (£) by sample type for targeted PCR positive results at a London NHS laboratory network, April 2015 and April 2019.

Sample referred	Number sent	Cost(£)/positive targeted PCR	Cost(£)/positive targeted PCR and negative culture
Pus	18	317.94	317.94
CSF	62	410.67	410.67
Tissue/Biopsy Culture	49	324.56	370.93
Joint Fluid	40	264.95	302.80
Pleural Fluid	31	182.52	182.52
Bronchial Alveolar Lavage	6	79.49	79.49
Other	4	N/A	N/A
All samples	210	278.20	292.84

Abbreviations: ribosomal Ribonucleic Acid (rRNA), Polymerase Chain Reaction (PCR), Cerebrospinal Fluid (CSF).

## Discussion

Our study finds 16S PCR to be a useful but notably expensive test for bacterial detection in culture-negative samples in an NHS laboratory services network where third party reference laboratories are used for molecular diagnostics. Targeted PCR is cheaper per-positive result but still incurs a significant cost. Pus samples are the sample type of choice to produce a positive 16S PCR result, and respiratory fluids most likely to yield a positive result with targeted PCR. A large number of culture-positive samples were referred for 16S PCR testing, representing inefficient, inappropriate referral pathways. Molecular diagnostics have a small but contributory role in providing supportive or new microbiological diagnoses and assisting clinicians in their choice of antimicrobial therapy. Finally, the cost-per-positive of 16S PCR and targeted PCR is significantly different based on the sample type referred, with evidence to suggest targeted PCR is the more cost-effective.

In our study, 55 microbiological samples were culture-negative yet 16S PCR positive and 38 samples were culture-negative yet targeted PCR positive; when considering these samples are often from hospitalized patients, this offers significant potential to make clinically impactful decisions. Furthermore, the laboratory service network serves fifteen tertiary referral speciality services, where a microbiological diagnosis can often be critical to ongoing care. Rampini *et al*. demonstrated a 42.9% sensitivity of 16S PCR in culture-negative samples^16^ and Harris *et al*. demonstrated a 62% bacterial detection rate (43/69) by 16S PCR on heart valves from clinically confirmed infective endocarditis cases with culture-negative samples^10^. In our wider and more diverse clinical setting we found a lower rate of positivity of 9.9%, after discounting culture-positive samples. Furthermore, differentiating by sample type, we show 16S PCR utility to be significantly better with pus samples (including promisingly 7/12 brain abscesses). The low level of positive 16S PCR results from CSF was particularly notable, and we question the use of this test in this sample type. Tkadlec *et al*. report consistent data; of the 66 CSF samples tested only 6 additional positive results (9.1%) were returned by 16S PCR where culture was negative^9^. A meta-analysis conducted by Srinivasan *et al*. evaluated culture-negative CSF data from 15 studies and found 30% of cases yielded a positive result by 16S PCR, but a wide range between studies (3–100%) possibly reflecting the variation in inclusion criteria^11^. The sensitivity of 16S PCR for CSF samples is likely to depend on various factors from CSF volume, constituents (e.g. a pleocytosis) and extraction technique, which requires further evaluation for future standardisation.

Sample type was also of importance in detection rates for targeted PCR. Respiratory samples including BAL and pleural fluid had the highest yield for targeted PCR, predominantly demonstrating *Streptococcal pneumoniae*. With the introduction of rapid point of care/near patient multi-plex platforms for CSF samples, the use of 16S PCR in this setting may reduce and targeted PCR increase; our targeted bacteriology PCR analysis of CSF samples does however suggest questionable utility of this modality in this setting as well.

As shown by Morel *et al*., 16S PCR improves the diagnosis of fastidious organisms and assists in the identification in a wider array of organisms^7^. We show this is apparent when compared with targeted PCR. This benefit of 16S PCR over targeted PCRs should feed forward into the future construction of molecular testing algorithms (i.e. should a multiplex panel of likely organisms be undertaken first, and then only proceed to 16S PCR if this initial panel is negative?). Whilst not in routine clinical use, 16S PCR testing with deep sequencing, as opposed to Sanger sequencing, has proven an even more sensitive approach to diagnosing the flora of causative organisms in brain abscesses, and in particular their polymicrobial nature. Furthermore, Kommedal *et al*. demonstrated the presence of either or a combination of *Aggregatibacter aphrophilus*, *Fusobacterium nucleatum*, and *Streptococcus intermedius* in all spontaneous polymicrobial abscesses^20^. We do therefore urge a note of caution on the interpretation of 16S PCR results using Sanger sequencing and the limitations of low concentration bacteria isolates that ensues. The polymicrobial nature of certain infections also exposes the inherent limitation of targeted PCR; as the range of bacteria causing different clinical syndromes is constantly enlarging and changing, the repertoire of organisms ‘targeted’ from a sample will also need to evolve for a more certain microbiological diagnosis.

Whilst assessing the utility of molecular diagnostics, a clinically significant antimicrobial prescription change was observed in 15.4% of samples that had tested positive. O’Donnell *et al*. found a positive 16S PCR result supported the use of continued empiric antimicrobial therapy in 79% of patients and de-escalation of the current anti-microbial regimen in 21%, whilst escalation of antimicrobial therapy was not required for any positive PCR result^8^. We found molecular diagnostics helped in supporting or providing a new microbiological diagnosis in 79/109 clinical samples. This aids clinicians in antimicrobial choice, length of treatment, and overall clinical management. We do however report on a significant proportion of positive results which failed to impact clinical management as an alternate diagnosis was deemed more likely; this was more evident in 16S PCR positive results compared to targeted PCR and quite how to manage these errant molecular positive results remains a challenge.

The clinical significance of any result depends on timely processing and we report a turnaround-time of 13 days, a slight increase to that reported previously^8^. We therefore advocate that in an NHS laboratory network model, consideration be given to the practicalities and costs of repatriating tests such as these to ‘home-site’ laboratories rather than referral to third-parties. Laboratories with core-sequencing facilities typically have a turn-around time for 16S PCR and targeted PCR of 24–48 hours, reflecting the potential impact of repatriation. We propose further whole-healthcare-economy perspective cost-analyses be undertaken looking at the added value (or costs) arising from quicker turn-around-times as these molecular microbiological methods become more commonly used.

The cost-efficiency of molecular diagnostics relies on its employment in the setting of culture-negative samples. We found 51/607 samples referred had a culture-positive result (from prolonged incubation or subsequent specialist media), questioning the added utility of molecular diagnostics. Furthermore, the cost of 16S PCR/positive result rose from £400.78 to £568.37 when accounting for those results that were 16S PCR positive and culture-negative. We recommend SOPs restrict referral for 16S PCR until culture based methods have been exhausted. Additionally, the over 5.6 fold difference in cost-effectiveness between pus and CSF samples should mandate that sample type be strongly considered in SOPs prior to 16S PCR use. Additionally, targeted PCR is a cheaper test when employed, at a cost/positive result of £278.20, which rises to an average cost of £292.84 when culture-positive samples are excluded. Here, once again CSF samples are the most expensive. We therefore recommend targeted PCR be more commonly employed to assist microbiological diagnosis in culture-negative samples, especially when considering referral of respiratory samples.

Our study does have some limitations. The retrospective collection of clinical impact and prescribing data can mean the subtleties in interpretation of the molecular results at the time of the receipt may have been overlooked. NWLP refers to three third-party laboratories for bacterial molecular diagnostics which may reflect a varying sensitivity/specificity from their testing methods, masked in the aggregate data. Furthermore, molecular diagnostics should be ideally performed on sterile site samples; BAL samples, when taken from hospitalised patients can have a reduced specificity due to prolonged intubation and variation in prior port cleaning, which was not controlled for in our study^21^.

## Conclusions

16S PCR and targeted PCR demonstrate clinical utility in aiding bacterial identification in a range of clinical specimens, but particularly in pus and respiratory samples respectively. This extends to assisting in antimicrobial choice in a limited number of cases. We find a wider array of microorganisms identified with 16S PCR compared to culture and targeted PCR, and suggest molecular testing algorithms be constructed accordingly. The cost-effectiveness of 16S PCR is improved when strictly culture-negative samples are referred, with a marked variability in cost-per-positive-result between sample types with both molecular diagnostic techniques. We recommend laboratory SOPs be adjusted to reflect these findings, generating potential clinical benefit and cost-savings for healthcare facilities.

### List of abbreviations

Polymerase Chain Reaction (PCR); standards of microbiology investigations (SMI); ribosomal ribonucleic acid (rRNA); National Health Service (NHS); North-west London Pathology (NWLP); standard operating procedures (SOPs); European committee on antimicrobial susceptibility testing (EUCAST); bronchioalveolar lavage (BAL)

## Supplementary information

Supplementary information.

## Data Availability

The datasets analysed during the current study and further details on gaining access to the intervention reported within this study are available from the corresponding author (LSPM; l.moore@imperial.ac.uk) on reasonable request, as long as this meets local ethics and research governance criteria.
