# Supplementary material for: Clinical utility and cost-effectiveness of bacterial 16S rRNA and targeted PCR based diagnostic testing in a UK microbiology laboratory network
Source: Sci Rep. 2020 May 14;10:7965. doi: 10.1038/s41598-020-64739-1 (PMC7224368; doi:10.1038/s41598-020-64739-1)
Supplement: Supplementary file 1 — Supplementary information. [file 41598_2020_64739_MOESM1_ESM.docx]

**Clinical utility and cost-effectiveness of bacterial 16S rRNA and targeted PCR based diagnostic testing in a UK microbiology laboratory network.**

Dinesh Aggarwal^1,2^ dinesh.aggarwal@nhs.net

Tanmay Kanitkar^1^ t.kanitkar@nhs.net

Michael Narouz^3^ michael.narouz13@imperial.ac.uk

Berge Azadian ^1,3^ berge.azadian@nhs.net

*Luke SP Moore^1,2,3^ luke.moore@nhs.net

Nabeela Mughal^1,2,3^ nabeela.mughal@nhs.net

**Affiliations:**

1. Chelsea and Westminster NHS Foundation Trust, 369 Fulham Road, London, SW10 9NH. UK.

2. North West London Pathology, Fulham Palace Road. London. W6 8RF. UK.

3. Imperial College London, Hammersmith Campus, Du Cane Road, London. W12 0NN. UK

***Corresponding author:**

Dr Luke SP Moore, National Institute for Health Research Health Protection Research Unit in Healthcare Associated Infections and Antimicrobial Resistance, Imperial College London, Hammersmith Campus, Du Cane Road, London, W12 0NN, UK. Email: l.moore@imperial.ac.uk Telephone: 020 3315 8273

Supplementary Table 1: Diversity and results of tissue samples sent for 16S rRNA PCR and targeted PCR results at a London NHS laboratory network, April 2015 and April 2019.

|  | Targeted PCR | | 16S PCR | |
| --- | --- | --- | --- | --- |
| Tissue Type | **Number** | **Positive** | **Number** | **Positive** |
| Abdominal mass | 1 | 0 | 2 | 0 |
| Axillary mass | 0 | 0 | 1 | 1 |
| Bone | 13 | 1 | 36 | 2 |
| Brain | 5 | 1 | 17 | 2 |
| Discitis | 1 | 0 | 4 | 0 |
| Joint - native | 1 | 0 | 4 | 0 |
| Joint - prosthetic | 1 | 0 | 13 | 1 |
| Lung | 0 | 0 | 1 | 1 |
| Lymph node | 6 | 2 | 6 | 0 |
| Mediastinum | 0 | 0 | 1 | 0 |
| Muscle | 0 | 0 | 1 | 0 |
| Pacemaker | 0 | 0 | 2 | 0 |
| Paraspinal | 1 | 0 | 1 | 0 |
| Pericardium | 0 | 0 | 1 | 0 |
| Placenta | 0 | 0 | 1 | 0 |
| Pleural | 1 | 0 | 1 | 0 |
| Skin | 4 | 1 | 5 | 0 |
| Spleen | 0 | 0 | 1 | 0 |
| Thrombus | 0 | 0 | 1 | 0 |
| Valve - native | 14 | 3 | 23 | 5 |
| Valve - prosthetic | 0 | 0 | 1 | 0 |
| Vascular graft | 0 | 0 | 4 | 2 |
| Vasectomy | 0 | 0 | 1 | 0 |
| Unknown | 1 | 0 | 3 | 0 |
| Total | **49** | **8** | **131** | **14** |

Abbreviations: ribosomal ribonucleic acid (rRNA); Polymerase Chain Reaction (PCR)

Supplementary Table 2: Diversity and results of pus samples sent for 16S rRNA PCR and targeted PCR results at a London NHS laboratory network, April 2015 and April 2019.

|  | Targeted PCR | | 16S |  |
| --- | --- | --- | --- | --- |
| Pus site | **Number** | **Positive** | **Number** | **Positive** |
| Abdominal abscess | 1 | 0 | 3 | 2 |
| Aortic root abscess | 0 | 0 | 2 | 0 |
| Axillary abscess | 0 | 0 | 1 | 0 |
| Bone | 0 | 0 | 4 | 0 |
| Brain abscess | 3 | 0 | 12 | 7 |
| Breast implant | 0 | 0 | 1 | 0 |
| Chest Lump | 0 | 0 | 1 | 1 |
| Discitis | 2 | 0 | 5 | 0 |
| Groin abscess | 1 | 0 | 4 | 1 |
| Head superficial abscess | 0 | 0 | 4 | 0 |
| Joint - native | 1 | 1 | 1 | 1 |
| Limb collection | 1 | 1 | 2 | 2 |
| Liver abscess | 0 | 0 | 7 | 3 |
| Lung abscess | 0 | 0 | 1 | 1 |
| Lymph node abscess | 1 | 1 | 2 | 0 |
| Mediastinal collection | 1 | 0 | 1 | 0 |
| Nasal aspirate | 0 | 0 | 1 | 1 |
| Neck abscess | 2 | 0 | 6 | 5 |
| Nephrostomy pus | 0 | 0 | 2 | 0 |
| Otitis media | 0 | 0 | 1 | 0 |
| Ovarian abscess | 0 | 0 | 2 | 1 |
| Psoas abscess | 0 | 0 | 2 | 1 |
| Renal abscess | 0 | 0 | 4 | 1 |
| Spinal abscess | 3 | 0 | 6 | 1 |
| Uterine abscess | 0 | 0 | 1 | 0 |
| Vascular graft | 0 | 0 | 1 | 0 |
| Wound pus | 0 | 0 | 2 | 1 |
| Unknown | 2 | 0 | 5 | 0 |
| Total | **18** | **3** | **84** | **29** |

Abbreviations: ribosomal ribonucleic acid (rRNA); Polymerase Chain Reaction (PCR)
